# Increased Cell Proliferation and Gene Expression of Genes Related to Bone Remodeling, Cell Adhesion and Collagen Metabolism in the Periodontal Ligament of Unopposed Molars in Growing Rats

**DOI:** 10.3389/fphys.2017.00075

**Published:** 2017-02-10

**Authors:** Domna Dorotheou, Vassiliki Farsadaki, Marie-Luce Bochaton-Piallat, Catherine Giannopoulou, Thanos D. Halazonetis, Stavros Kiliaridis

**Affiliations:** ^1^Department of Orthodontics, University of GenevaGeneva, Switzerland; ^2^Department of Pathology and Immunology, University of GenevaGeneva, Switzerland; ^3^Department of Periodontology, University of GenevaGeneva, Switzerland; ^4^Department of Molecular Biology, University of GenevaGeneva, Switzerland

**Keywords:** tooth eruption, gene expression, periodontal ligament, dental occlusion, cell proliferation

## Abstract

Tooth eruption, the process by which teeth emerge from within the alveolar bone into the oral cavity, is poorly understood. The post-emergent phase of tooth eruption continues throughout life, in particular, if teeth are not opposed by antagonists. The aim of the present study was to better understand the molecular processes underlying post-emergent tooth eruption. Toward this goal, we removed the crowns of the maxillary molars on one side of the mouth of 14 young rats and examined gene expression patterns in the periodontal ligaments (PDLs) of the ipsilateral and contralateral mandibular molars, 3 and 15 days later. Nine untreated rats served as controls. Expression of six genes, Adamts18, Ostn, P4ha3, Panx3, Pth1r, and Tnmd, was upregulated in unopposed molars relative to molars with antagonists. These genes function in osteoblast differentiation and proliferation, cell adhesion and collagen metabolism. Proliferation of PDL cells also increased following loss of the antagonist teeth. Interestingly, mutations in PTH1R have been linked to defects in the post-emergent phase of tooth eruption in humans. We conclude that post-emergent eruption of unopposed teeth is associated with gene expression patterns conducive to alveolar bone formation and PDL remodeling.

## Introduction

The process, by which teeth emerge from within the alveolar bone into the oral cavity, is referred to as tooth eruption and is divided in two discernible phases: the pre-emergent phase, when the tooth is still in its bony crypt, and the post-emergent phase, which starts when the tooth penetrates the oral mucosa. The mechanisms underlying tooth eruption are still being investigated, yet it already appears that distinct processes operate during the pre- and post-emergent phases (Sarrafpour et al., 2013).

Here, we focused our efforts on the post-emergent phase of tooth eruption. Several studies have shown that this phase is stimulated by loss of the antagonist teeth. One cross-sectional study examined the records of 53 adult individuals having molars without antagonists for at least 10 years and recorded mild overeruption (≤2 mm) in 76% of unopposed molars (Kiliaridis et al., 2000). In another cross-sectional study, 92% of teeth without antagonists presented with overeruption (Craddock et al., 2007). In these two studies, overeruption was more pronounced in the maxillary, as compared to the mandibular, teeth and also in the presence of periodontal inflammation. Post-emergent tooth eruption has also been investigated in longitudinal studies. A group of adult individuals with unopposed maxillary molars was followed over a 10-year period; vertical displacement of the unopposed teeth during this period was two times greater than that of the teeth with antagonists (Christou and Kiliaridis, 2007). Nevertheless, overeruption did not lead to clinical problems in most of these individuals. However, in another study focusing on children and adolescents, loss of the upper second molars resulted, over a 10-year period, in clinically significant overeruption of the unopposed lower permanent molars in all cases (Smith, 1996). Thus, loss of antagonist teeth and age seem to be important factors influencing post-emergent tooth eruption in humans.

Experimental animal models, notably rats, have also been used to study post-emergent eruption of unopposed teeth. In these models, occlusal contacts were eliminated either by extracting the antagonist teeth or by grinding away the tooth crowns. As in humans, the unopposed teeth overerupted and the degree of vertical displacement was more pronounced in younger animals, than in adults (Deporter et al., 1982; Kinoshita et al., 1982; Fujita et al., 2009).

In the animal models, antagonist tooth loss and the resulting post-emergent tooth eruption are associated with changes in the histology of the PDL. First, there is a reduction in the number, diameter and mineral density of Sharpey fibers of the unopposed teeth (Short and Johnson, 1990). Sharpey fibers are the collagen fibers that penetrate the surface layers of the alveolar bone and of the cementum covering the roots of the teeth. These fibers are primarily responsible for resisting any extrusive forces acting on the teeth. Thus, the reduction in their number and diameter might weaken tooth support, allowing eruption (Short and Johnson, 1990). The tensile strength of the PDL is also reduced when eruption is induced due to antagonist removal. A possible explanation could be the changes in the Sharpey fibers mentioned above, plus continuous remodeling of the periodontium, resulting in loosening of the attachment of the periodontal fibers to the alveolar bone (Kinoshita et al., 1982).

In addition to the changes in the Sharpey fibers, post-emergent tooth eruption is associated with changes in the anatomy of the PDL, including narrowing of the periodontal space (Cohn, 1966), vascular constriction (Watarai et al., 2004) and deformation of the mechanoreceptor structure of the PDL (Muramoto et al., 2000). The structure and metabolism of the collagen and extracellular matrix of the PDL are also affected by loss of an antagonist tooth (Johnson, 1989; Afanador et al., 2005). However, it is not yet clear, if the alterations cited above are causatively linked to the process of tooth eruption or an adaptation to reduced tooth function.

Some of the genes possibly involved in the signaling cascades of tooth eruption have been proposed like *Adamts18* which is involved in extracellular matrix turnover in the PDL (Song et al., 2013) and has been linked to increased blood flow (Wei et al., 2014). Another gene is *Panx3* which promotes osteoblast differentiation (Ishikawa et al., 2014) as well as *Ostn*, which is produced by osteoblasts and is involved in osteoblast differentiation and proliferation (Bocciardi et al., 2007; Moffatt and Thomas, 2009).

*Pth1r*, Parathyroid hormone 1 receptor, has been linked to primary failure of eruption (PFE), a tooth eruption disorder, of the post-emergent phase of tooth eruption (Proffit and Vig, 1981). *Tnmd* is another gene that was found to be related to the function and maturation of the PDL at the time when tooth muscticatory function occurs (Komiyama et al., 2013).

In an attempt to gain further insights into the molecular mechanisms underlying post-emergent tooth eruption, we monitored gene expression in the PDL of unopposed molars using a rat model system. We found significant changes in expression only for a handful of genes. Remarkably, mutations in one of these genes, PTH1R, lead to tooth eruption failure in humans.

## Materials and methods

### Ethical approval

The experimental protocol was approved by the General Direction of Health, Domain of Animal Experiments, Canton of Geneva, Switzerland. CN: 1080/3807/2.

### Experimental design

**Figure 1:**
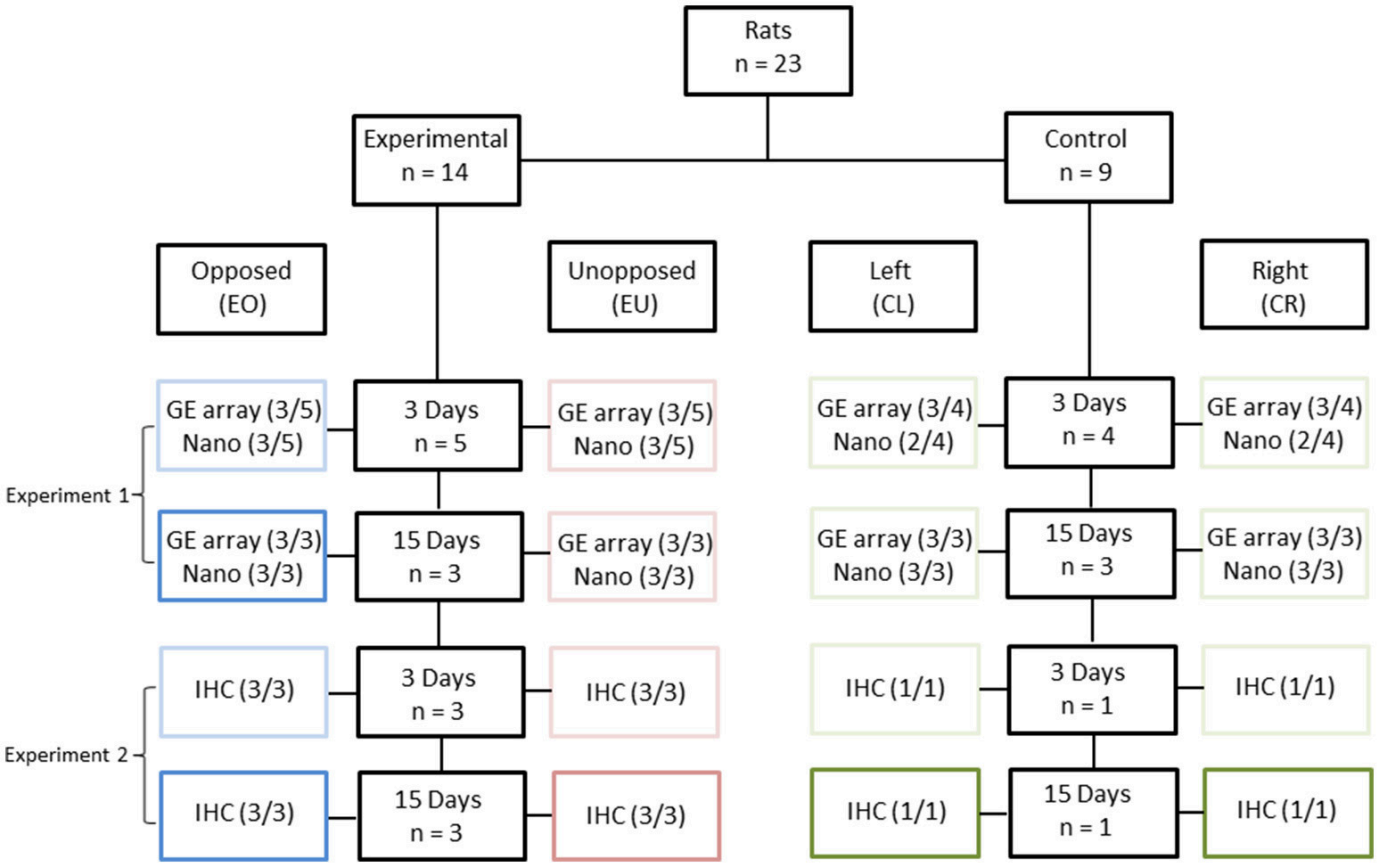
**Experimental design**. Twenty three rats were divided into two groups [Experimental (E), *n* = 14 and Control (C), *n* = 9], based on the presence of unopposed molars. In the control groups, right (CR) and left (CL) molars were considered as one pool of teeth with antagonists. The experimental periods were 3 days (3) and 15 days (15). The right maxillary molar crowns of 14 experimental animals were cut down, leading to unopposed molars at the right side (EU), in contrast to the molars of the left side which were opposed by their antagonists (EO). Periodontal ligament samples were distributed across the following three experiments: Experiment 1: Gene Expression microarray (GE array) and Nanostring (Nano). Three out of three samples (3/3) were used in this experiment, except for the Nanostring-analyzed 3-day groups, where only one of three samples (1/3) was used, complemented by Experiment 2. Experiment 2: Nanostring. Two out of two (2/2) and one out of one (1/1) samples were used for the experimental and control groups, respectively, as complementary to Experiment 1. Experiment 3: Immunohistochemistry (IHC). Three out of three (3/3) and one out of one (1/1) samples were used for the experimental and control groups, respectively.

Twenty-three, 4-week old, male Wistar rats were used in this study: 14 in the experimental group and nine in the control group (Figure 1). In the experimental group, the crowns of the right maxillary molars were removed following anaesthesia, while in the control group no dental intervention took place. In the experimental group, the right mandibular molar had no occlusion during the whole experimental period. The PDLs of the right and left, first mandibular molars (two teeth per animal) were then studied, either 3 or 15 days after crown removal. In total, 46 teeth were examined and these were assigned to eight conditions, as follows:

- Experimental animals/Unopposed molars (right mandibular molars)/3 or 15 days after crown removal (EU3 and EU15, respectively);
- Experimental animals/Opposed molars (left mandibular molars)/3 or 15 days after crown removal (EO3 and EO15, respectively);
- Control animals/Right mandibular molars/4-week and 3-day old or 6-week old animals (CR3 and CR15, respectively);
- Control animals/Left mandibular molars/4-week and 3-day old or 6-week old animals (CL3 and CL15, respectively).

The unopposed molars in the experimental animals were considered to be hypofunctional; the opposed molars were probably hyperfunctional due to the higher masticatory demands placed on them; while the left and right molars in the control animals should have been subjected to physiological masticatory demands. Since the left and right molars of the control animals could be grouped together, the number of control animals could be lower than the number of experimental animals, thereby minimizing animal suffering (Burden et al., 2015).

Two series of experiments were performed, in order to obtain the material needed for this study.

(a) At the end of the first experiment the first mandibular molars (right and left) were extracted from 15 animals, eight experimental and seven controls. Nine animals, five experimental and four controls were sacrificed 3 days after having cut the maxillary molars and six animals, three experimental and three controls 15 days after having cut the maxillary molars. The PDL was carefully scraped with a scalpel from each root of the molar and RNA extraction was performed. Thus, the RNA from the 30 PDL tissues was used for cDNA microarray and nanostring analysis. (b) In the second experiment we used eight animals, six experimental and two controls for immunohistochemical (IHC) analysis. Four rats, three experimental and one control were sacrificed 3 days and the rest four rats (three experimental and one control), 15 days after having cut the right maxillary molars. Each one of these eight rats received an intraperitoneal single pulse injection of 40 mg/kg BrdU (bromodeoxyuridine) (B5002, Sigma, St. Louis, MO, USA) 1 day before sacrifice. Their mandibles were dissected, fixed, decalcified, dehydrated and embedded in paraffin for further immunohistochemical analysis.

During the experimental period the animals were fed with a soft diet and water *ad libitum*. The day/night rhythm was ensured by automatic dimmed lighting (08:00–20:00 h). Body weight measurements were acquired every 2 days and served as an indicator of the general physical condition of the animals.

### RNA isolation

Total RNA was extracted using the RNeasy Mini Kit (Qiagen, Hilden, Germany), according to the manufacturer's instructions. The extracted RNA was eluted in 30 μl RNase-free sterile water (provided with the kit). Half of the isolated RNA was used for complementary DNA (cDNA) microarray experiments and the other half for Nanostring nCounter Expression Analysis.

### cDNA microarray transcriptome profiling

Transcriptome profiling was performed using Affymetrix Gene Chip RaGene 2.0 st v1 arrays (Santa Clara, CA, USA), according to the instructions of the manufacturer. Three independent biological replicates were processed per condition, resulting in a total of 24 samples. The quality and quantity of RNA were assessed using a Bioanalyzer (model 2100 with RNA 6000 Nano Chips, Agilent Technologies, Amstelveen, The Netherlands). Two hundred Nano gram of total RNA from each sample was used to prepare double-stranded cDNA, from which biotin-labeled cRNA was synthesized using the BioArray HighYield RNA Transcript Labeling Kit (Enzo). After purification on a QIAGEN RNeasy column, the cRNA was fragmented and hybridized to the arrays. Hybridization, washing and scanning of the arrays were performed according to the manufacturer's instructions. The image data were processed using Affymetrix MAS 5.0 software to generate gene expression data, which were normalized using a robust multi array (RMA) protocol (Bolstad et al., 2003) and then assessed for statistical significance using a 3-way ANOVA test implemented in the Partek Genomic Suite (http://www.partek.com).

### Nanostring gene expression analysis

Twenty-two RNA samples prepared from PDLs isolated in the first and second series of experiments were processed for Nanostring analysis. Samples corresponding to conditions CR3 and CL3 were considered equivalent, as were samples corresponding to conditions CR15 and CL15. This reduced the number of conditions to six: EO3, EU3, CR3/CL3, EO15, EU15, and CR15/CL15. For each condition, at least three independent, biological replicates were processed.

Expression of 53 genes, 47 experimental and 6 normalization genes was examined using the nCounter system (NanoString). Selection of these genes was based on the transcriptome data obtained by the microarray analysis and on biological insights. The expression of the normalization genes was similar among all groups studied. For each sample, 200 ng of total RNA was hybridized with multiplexed Nanostring probes, as described previously (Geiss et al., 2008). Background correction was done by subtracting from the raw counts the mean +2 standard deviations of counts obtained with the negative controls. Values <1 were fixed to 1. Positive controls were used for quality assessment: the ratio between the highest and the lowest positive controls average among samples was below 3. Then counts for target genes were normalized with the geometric mean of the 6 normalization genes selected as the most stable using the geNorm algorithm (Vandesompele et al., 2002). Statistical analysis (3-way ANOVA) of the data was performed using the Partek Genomics Suite (http://www.partek.com).

### Immunohistochemical staining

Sixteen semi-mandibles were dissected for immunohistochemical analysis. Three semi-mandibles were used for each of the following conditions: EO3, EU3, EO15, EU15 and one semi-mandible was used for each of the control conditions: CR3, CL3, CR15, and CL15. The mandibles were fixed in 4% paraformaldehyde (Merck 8.18715, Darmstadt, Germany) for 2 days, decalcified with a solution of 15% EDTA [pH 7.4] and 0.5% paraformaldehyde for 12 weeks, embedded in paraffin, and then sectioned at the frontal plane with a thickness of 3 μm. Paraffin-embedded tissue sections were subjected to deparaffinization and hydration. Endogenous peroxidase activity was then blocked with EnVision Flex Peroxidase-Blocking Reagent (Dako North America, Carpinteria, CA, USA) for 10 min, after which the sections were washed three times with EnVision Flex Wash Buffer (DAko), for 5 min each, followed by denaturation for 20 min in a solution of denaturation (HCL/Triton). Borax (N. 33648, Sigma) treatment was followed for 30 min. The sections were washed three times with EnVision Flex Wash Buffer (DAko), for 5 min each. The sections were then incubated overnight with the primary antibody (anti-BrdU mouse monoclonal antibody; Cat. No. 11170376001, Roche) diluted 1:50 in EnVision Flex Antibody diluent (Daco). The sections were washed three times with EnVision Flex Wash Buffer (DAko), for 5 min each. Incubation with the secondary antibody was followed, (EnVision+ Flex/HRP (K8024, Dako) for 30 min, in room temperature. EnVision Flex DAB (Dako) was used for color development according to the manufacturer's instructions. The sections were counterstained with hematoxylin (No. 1092532500, Merck). Negative control sections were treated in the same manner, except that primary antibody was not added.

For the quantification of BrdU-positive cells, the apical part of the PDL of the first mandibular molars was imaged using a Zeiss (Axio Scan.Z1) microscope in two different areas, vestibular and lingual. Both areas were imaged at 10x and 40x magnification in 8-bit tiff pictures. In order to avoid double-counting of cells, 3 non-consecutive sections with at least 12 μm distance between each section were evaluated from each semi-mandible. A total of 96 images were taken arbitrarily in this region with 40x magnification, 18 from each experimental group (EO3, EU3, EO15, and EU15) and 6 from each control group (CR3, CL3, CR15, and CL15). The total number of cells and the number of positively BrdU-stained cells were measured. The percentage of BrdU-positive cells for each group was calculated and is presented in **Figure 4C**.

## Results

The right and left mandibular molars from 23 animals were placed into 8 groups, as described above, based on the presence or absence of antagonist molars, and according to the length of the experimental period, namely 3 or 15 days. In all of the analyses, the results of the right and left molars of the control groups were pooled.

### Gene expression profiles

From the 28,407 RefSeq transcripts covered by the Affymetrix Gene Chip 2.0, 700 genes presented more than a 2-fold increase or decrease in expression between the experimental and control groups. From this list, 47 genes were selected for further analysis based on their possible relevance to tooth eruption: *Adam12, Adamts18, Alpl, Bmp3, Col12a1, Col6a3, Cpz, Dchs1, Ednra, Fat4, Fkbp10, Fkbp14, Fmod, Fndc3b, Fzd2, Grb10, Gtpbp4, Igsf10, Itga11, Lox, Mab21l2, Mdk, Mmp2, Mmp9, Myh10, Myl9, Mylk, Ncam1, Ostn, P4ha3, Panx3, Pcolce, Plod2, Prickle1, Prrx1, Pth1r, S100g, Sfrp4, Slc16a7, Tagln, Thbs2, Tmem119, Tnfrsf11b, Tnmd, Tnn, Vcan, and Zfp354c* (Table 1 in Supplementary Materials).

To confirm that the expression of these 47 genes differed between the experimental and control groups, we monitored their expression by a second method, referred to as Nanostring, that detects and counts single mRNA molecules. For this analysis, the genes *Hprt1, Hsp90ab1, Nono, Ppih, Sdha*, and *Tbp* were used for normalization. The Nanostring analysis included additional biological replicates (Figure 1) and, thus, in addition to validating the microarray data, served to increase the sample size. Thus, from the 47 initially selected genes, expression of six genes, *Panx3, Ostn, Adamts18, Tnmd, Pth1r*, and *P4ha3*, correlated best with tooth eruption, when taking into account the data from both the microarray and Nanostring experiments (Table 2 in Supplementary Materials).

**Figure 2:**
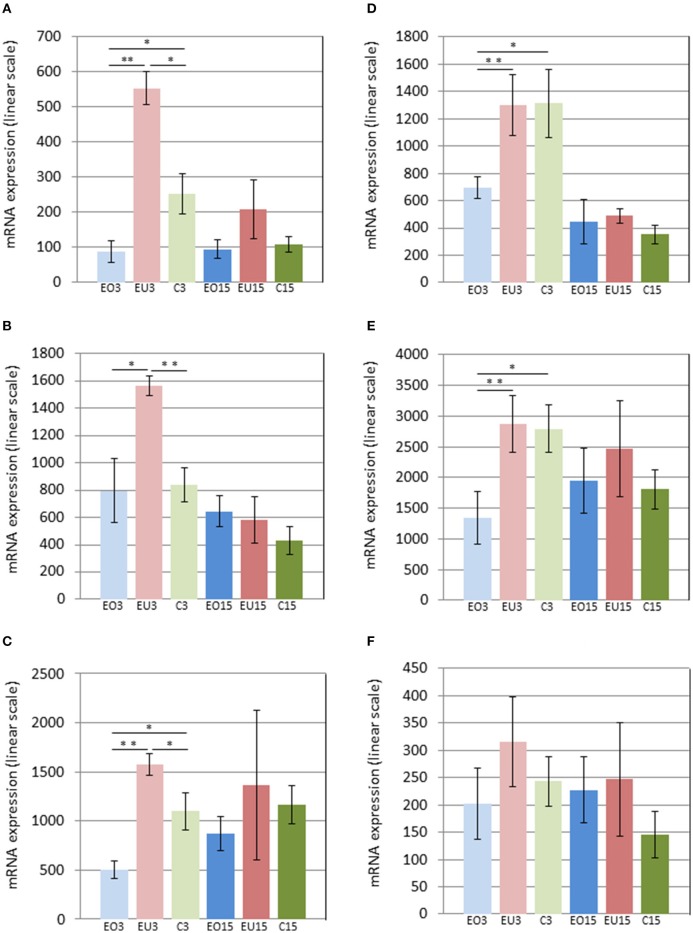
**mRNA levels in the PDL of first mandibular molars, as determined by Nanostring for the following genes: Panx3 (A)**, Ostn **(B)**, Adamts18 **(C)**, Tnmd **(D)**, Pth1r **(E)**, and P4ha3 **(F)**. EO3, Experimental Opposed at 3 days; EU3D, Experimental Unopposed at 3 days; C3, Control at 3 days; EO15, Experimental Opposed at 15 days; EU15, Experimental Unopposed at 15 days; C15, Control at 15 days. Mean gene expression is presented in arbitrary units. The data are presented in linear scale. The bars denote standard error of the mean and asterisks denote significant differences between groups (^*^P < 0.05; ^**^P < 0.01).

**Figure 3:**
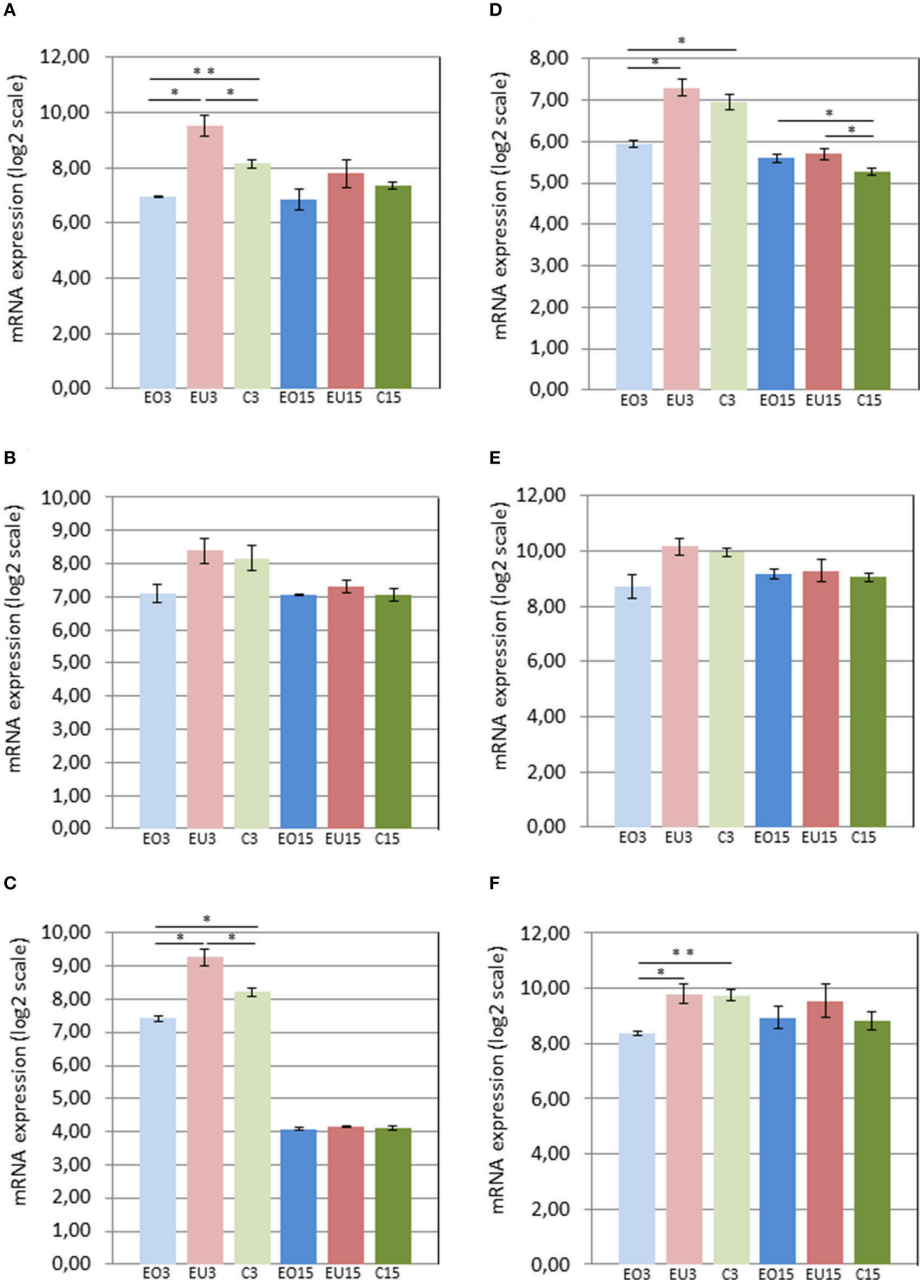
**mRNA levels in the PDL of first mandibular molars, as determined by cDNA microarray for the following genes: Panx3 (A)**, Ostn **(B)**, Adamts18 **(C)**, Tnmd **(D)**, Pth1r **(E)**, and P4ha3 **(F)**. EO3, Experimental Opposed at 3 days; EU3D, Experimental Unopposed at 3 days; C3, Control at 3 days; EO15, Experimental Opposed at 15 days; EU15, Experimental Unopposed at 15 days; C15, Control at 15 days. Mean gene expression is presented in arbitrary units. The data are presented in log2 scale. The bars denote standard error of the mean and asterisks denote significant differences between groups (^*^P < 0.05; ^**^P < 0.01).

Three days after removing the crowns of the maxillary molars in the experimental group, *Panx3* and *Adamts18* were upregulated in the PDL of the unopposed molars compared to the molars in the control group and downregulated in the opposed molars compared to the control teeth. At 15 days after crown removal, *Panx3* and *Adamts18* expression was also elevated in the PDL of the experimental unopposed molars and suppressed in the opposed molars, as compared to the control teeth, but these differences were more modest than the ones observed at 3 days and did not reach statistical significance (Figures 2A,C). The cDNA microarray analysis yielded similar results (Figures 3A,C).

The third gene examined, *Ostn*, was upregulated in the PDL of unopposed molars compared to opposed and control molars from the 3-day samples analyzed by Nanostring (Figure 2B). A similar expression pattern was true of *Pth1r*, which was also upregulated in unopposed vs. opposed molars from 3-day samples (Figure 2E). However, contrary to *Ostn, Pth1r* expression showed no difference between experimental unopposed and control molars across all samples. cDNA microarray results from 3-day samples confirmed the tendency observed for each gene, although not with statistical significance (Figures 3B,E).

Like *Pth1r, Tnmd*, the fifth gene examined, showed equal expression in experimental unopposed and control molars in 3-day samples, which was significantly greater than its expression in experimental opposed molars (Figure 2D). Further, this pattern was statistically significant in cDNA microarray analysis (Figure 3D). Interestingly, *Tnmd* expression was significantly upregulated in both experimental opposed and unopposed molars, vs. control, in PDL examined by cDNA microarray 15 days after crown removal (Figure 3D).

For the last gene examined, *P4ha3*, the only significant difference was observed in cDNA microarray analysis of 3-day samples, whereby unopposed and control molars both showed upregulated expression relative to opposed molars (Figure 3F).

### Immunohistological staining

**Figure 4:**
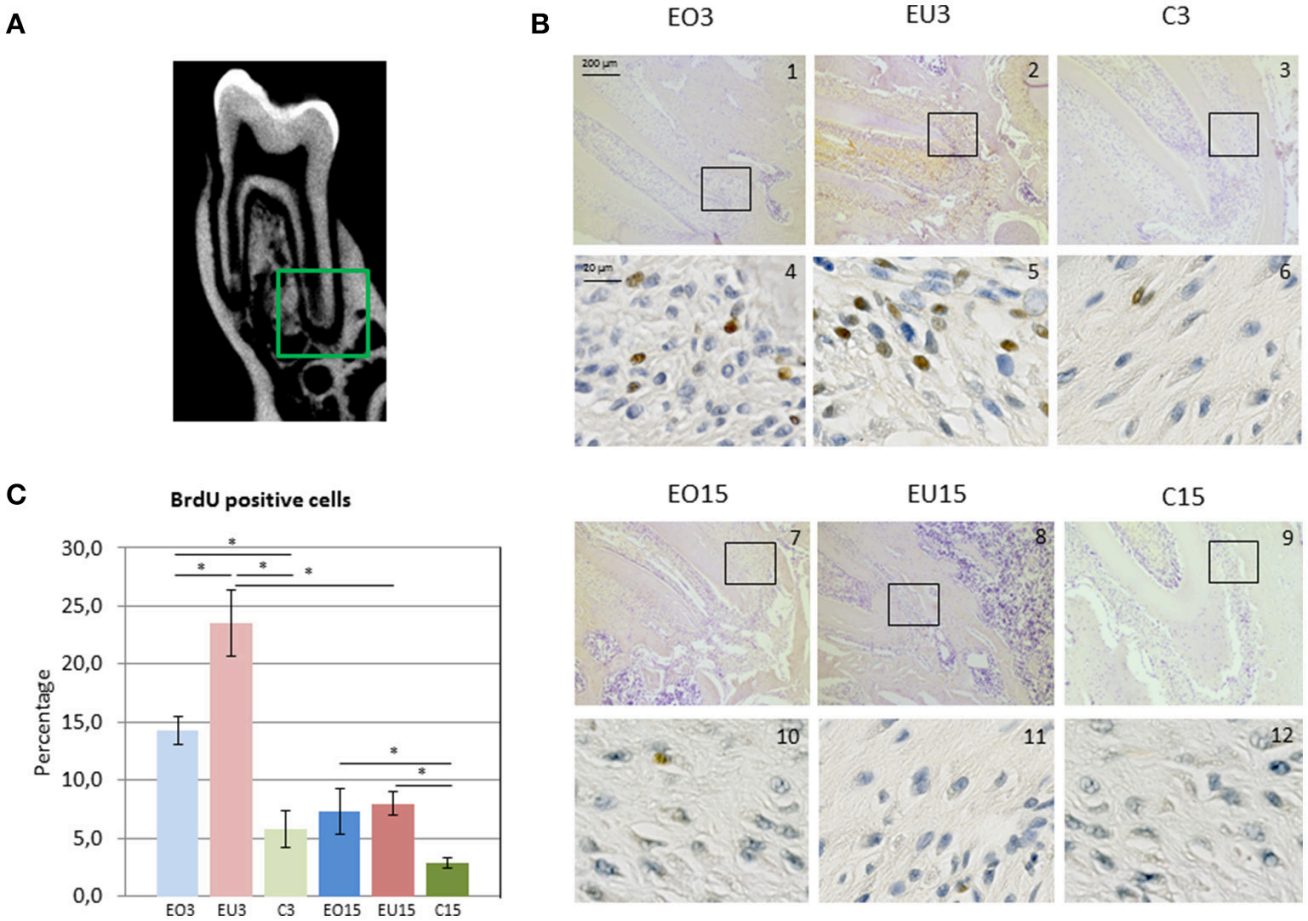
**(A)** Imaging of the 3 rat mandibular molars as presented with cone beam computed tomography. In the green square is the apical part of the first root of the first mandibular molar, corresponding to the area presented in panel **(B)** in images 1–3 and 7–9. **(B)** Immunohistochemical BrdU staining in the PDL of the first mandibular molar. Images 1–3 and 7–9 are magnified 10x; and images 4–6 and 10–12 40x. Black squares indicate the area viewed at higher magnification (40x). **(C)** Mean percentage of BrdU positive cells. EO3, Experimental Opposed at 3 days; EU3D, Experimental Unopposed at 3 days; C3, Control at 3 days; EO15, Experimental Opposed at 15 days; EU15, Experimental Unopposed at 15 days; C15, Control at 15 days. The bars denote standard error of the mean and asterisks denote significant differences between groups (^*^P < 0.05).

PDL sections were observed and evaluated in the whole length of the root. Proliferation activity was most remarkable in the apical part, mainly in the experimental unopposed and opposed molars for the three- and 15-day samples (Figures 4A,B).

BrdU incorporation was more pronounced in the 3-day than in the 15-day samples. This reflects increased cellular proliferation during the first 3 days following antagonist removal to support eruptive tooth movement, which then decreased with time. In both 3- and 15-day samples, the percentage of BrdU-positive cells was higher in experimental (opposed and unopposed) than in control molars. However, 3-day samples showed significantly greater BrdU incorporation in the PDL of unopposed molars than in opposed molars, whereas 15-day samples showed similar levels for both.

## Discussion

Post-emergent tooth eruption is a multifactorial developmental process involving movement of existing tissues, as well as resorption and formation of new tissues coordinated by a complex set of genetic and metabolic events. In the present study, we have used the model of the unopposed rodent molar to investigate the genetic mechanisms involved in axial tooth movement during post-emergent eruption. Cellular proliferation in the PDL of teeth without antagonists was high and slowed down with time. *Panx3, Ostn*, and *Adamts18* genes were upregulated in the PDL tissue of molars without antagonists, and *Panx3, Adamts18, Tnmd, Pth1r*, and *P4ha3* genes were downregulated in molars with antagonists, receiving excessive masticatory forces. This supports the hypothesis that soon after loss of the antagonist tooth, periodontal ligament turnover and bone formation increase to support tooth eruption, whereas upon augmented force, the abovementioned process is suppressed.

### Adamts18

The first gene studied, *Adamts18*, codes for a protease that belongs to the family of disintegrins and metalloproteinases (ADAMs) with thrombospondin characteristics. The mechanisms underlying its function in different tissues remain unclear (Wei et al., 2014). *Adamts18* has been shown to play a role in extracellular matrix turnover in the PDL tissue of permanent teeth (Song et al., 2013). Its presence has also been linked to increased blood flow in the context of periodontal ligament-associated tooth eruption (Wei et al., 2014), in the post-emergent phase in particular (Proffit and Frazier-Bowers, 2009). In our study, Adamts18 was upregulated in the PDL of the experimental unopposed molars 3 and 15 days after antagonist removal, which may be linked to increased blood flow in the area of the PDL during eruption. Interestingly, *Adamts18* expression was remarkably reduced in the PDL of the experimental opposed group, which suggests that increased masticatory loads may cause vasoconstriction, and therefore reduced blood flow.

### Panx3

*Panx3* is a member of the chordate channel proteins identified by their homology to insect gap junction proteins. It is abundant in skin, cartilage, and bone (Penuela et al., 2014). *Panx3* promotes osteoblast differentiation and regulates the switch from proliferation to differentiation phase in osteoprogenitor cells (Ishikawa et al., 2014). It is a target gene of Runx2, a transcription factor specific for osteogenesis (Bond et al., 2011). In our study, *Panx3* was remarkably upregulated in the PDL of the experimental unopposed molars 3 days after antagonist tooth removal, which may be linked to increased osteoblast differentiation. This, in turn, would support alveolar bone formation during tooth eruption. In contrast, under higher masticatory load, *Panx3* was downregulated, leading to osteoblast differentiation arrest. The expression of Panx3 was similar in both experimental and control groups 15 days later.

### Ostn

*Ostn*, which binds the natriuretic clearance receptor, is a small secreted protein with homology to natriuretic peptides. It is produced by osteoblasts (Thomas et al., 2003), muscles, tendons, and ligaments (Moffatt et al., 2007). Besides its hormone-like properties, Ostn is expressed in osteoblasts and may be involved in autocrine regulation of osteoblast differentiation and proliferation (Bocciardi et al., 2007; Moffatt and Thomas, 2009). In our study, *Ostn* was upregulated in the PDL of unopposed molars 3 days after antagonist removal, which may therefore be linked to increased osteoblast differentiation and proliferation, ultimately leading to alveolar bone formation in the process of tooth eruption.

The observation that overall expression levels of *Panx3* and *Ostn* decreased remarkably with time, as shown by comparing results of the 3- and 15-day samples, indicate that osteoblast maturation, alveolar bone formation, and the larger part of the eruptive tooth movement most likely took place soon after the antagonist tooth removal.

### Pth1r

*Pth1r*, is expressed in forming bone but not in resorbing bone surfaces, mostly in osteocytes and in regions of net bone formation (Fermor and Skerry, 1995). This gene has been linked to a tooth eruption disorder known as PFE, which was first described by Proffit and Vig (1981). In PFE, a non-ankylosed tooth fails to erupt due to a disturbance in the eruption mechanism, mainly during the post-emergent phase. Non-syndromic PFE is an autosomal dominant disorder that is caused by heterozygous mutations of the *Pth1r* gene (Frazier-Bowers et al., 2010; Stellzig-Eisenhauer et al., 2010; Yamaguchi et al., 2011; Raberin et al., 2015), leading to protein haploinsufficiency (Roth et al., 2014). Mutations of *Pth1r* causing PFE have also been linked to osteoarthritis (Frazier-Bowers et al., 2014). Recently, a mutational overlap has been identified between Blomstrand chondrodysplasia and PFE (Risom et al., 2013). It has also been demonstrated that in PFE with *Pth1r* mutations, the affected teeth reabsorb coronally-located alveolar bone, but nevertheless do not erupt due to bone formation arrest (Pilz et al., 2014). We observed *Pth1r* to be almost equally expressed in the PDL of the experimental unopposed molars and control molars in the 3-day groups; however, it was less expressed in the experimental opposed group, indicating reduced bone apposition in case of higher masticatory load. However, there was no difference in expression of *Pth1r* between the experimental and control groups 15 days later. Insofar as normal eruption takes place during mandibular growth, our findings from the expression profile of Pth1r may indicate active bone formation, as well as overeruption due to antagonist removal. Contralaterally to the unopposed side, bone apposition arrest may occur as a result of increased masticatory loads.

### Tnmd

Tnmd is a transmembrane protein expressed in dense connective tissue such as ligaments and tendons and known to be upregulated in their late developmental stages (Shukunami et al., 2001). In previous studies, *Tnmd* expression was investigated in 2-, 3-, and 4-week-old mice. Its expression was first upregulated in 2-week-old mice during the pre-emergent phase of tooth eruption. It then became more pronounced in 3-week-old mice during the post-emergent phase before reaching the occlusal plane, and even more so in 4-week-old mice with synchronized masticatory function. *Tnmd* expression appears to be related to the function and maturation of the PDL by positively regulating fibroblast adhesion and collagen fibril maintenance in the PDL, at the time when tooth function first occurs (Komiyama et al., 2013). In our study, *Tnmd* was equally expressed in the PDL of 4-week-old experimental unopposed and control molars, 3 days after antagonist tooth removal. Its expression was downregulated in the experimental opposed molars, possibly due to increased masticatory forces. In the 15-day groups, *Tnmd* expression was reduced in the unopposed and control molars, possibly linked to PDL aging in the latter.

### P4ha3

*P4ha3* encodes a component of prolyl 4-hydroxylase, a key enzyme in collagen synthesis composed of two identical alpha subunits and two beta subunits. The encoded protein is one of several different types of alpha subunits and provides the major part of the catalytic site of the active enzyme. In collagen and related proteins, prolyl 4-hydroxylase catalyzes the formation of 4-hydroxyproline that is essential to the proper three-dimensional folding of newly synthesized procollagen chains. In our study, *P4ha3* was equally expressed in the unopposed and control molars and downregulated in the PDL of the experimental opposed molars 3 days after antagonist removal, showing collagen remodeling suppression in the PDL due to masticatory force excess. Its expression was also downregulated in the control molars of the 15-day groups relative to the 3-day groups. This may indicate possible alteration of collagen metabolism of the PDL with aging.

At this point it is worth noting that for the six genes we focused on, the levels of expression were generally lower at the 15 day time point, than at the 3 day time point (Figures 2, 3). This was true, even when expression of these genes in the PDLs of the control teeth was compared between the 3 and 15 day groups. We attribute this systematic difference to reduced cellularity and reduced thickness of the PDL at the 15 day time point, reflecting the difference in age and developmental stage of the animals. Thus, if one considers an equal number of contaminating cells (blood cells, etc.) in all PDL samples, a smaller fraction of the total RNA would be derived from PDL cells in the older animals. Irrespective of the reason underlying the lower expression in the 15 day samples, it is clear that the comparisons of gene expression should be limited to groups of the same time point (i.e., within the 3 day groups and within the 15 day groups).

## Conclusions

The mechanism of post-emergent tooth eruption remains largely unknown. In our study, we have shown that upon antagonist tooth removal and masticatory force alteration, gene expression of the periodontal ligament changes. In case of experimental unopposed molars, the *Adamts18, Panx3, Ostn, Pth1r*, and *Tnmd* genes are upregulated, indicating increased cell proliferation, blood flow, and bone formation, as well as collagen remodeling around the teeth. These physiological observations are in accordance with the biological process of axial tooth translocation through the alveolar bone. In the case of experimental opposed molars with high masticatory loads, the *Adamts18, Panx3, Pth1r*, and *Tnmd* genes are downregulated, suggesting a possible decline of the abovementioned physiological changes. We can hypothesize that an increased cellular proliferation and bone modeling of the alveolar process takes place at a faster pace soon after loss of the antagonist tooth, which slows down with time. This decelerate of the axial movement of the tooth may be due to the establishment of a new force equilibrium.

In conclusion, we present here a novel implication for *Adamts18, Panx3, Pth1r, Ostn, Tnmd*, and *P4ha3* in post-emergent tooth eruption of rat molars without antagonists. Further work will shed light on their individual roles and functional interplay in this process.

## Author contributions

Conceived and designed the experiments: DD, CG, TH, SK. Performed the experiments: DD. Analyzed the data: DD, VF, MB, TH, SK. Wrote the paper: DD, VF, TH, SK.

### Conflict of interest statement

The authors declare that the research was conducted in the absence of any commercial or financial relationships that could be construed as a potential conflict of interest.
