# Supplementary material for: Increased Cell Proliferation and Gene Expression of Genes Related to Bone Remodeling, Cell Adhesion and Collagen Metabolism in the Periodontal Ligament of Unopposed Molars in Growing Rats
Source: Front Physiol. 2017 Feb 10;8:75. doi: 10.3389/fphys.2017.00075 (PMC5301028; doi:10.3389/fphys.2017.00075)
Supplement: Supplementary file 1 [file Table1.PDF]

TABLE S1: Results of microarray analysis. Log2 values of the mean and standard error of the raw data.

	3 DAYS						15 DAYS					
	EO		EU		C		EO		EU		C	
	MEAN	SE	MEAN	SE	MEAN	SE	MEAN	SE	MEAN	SE	MEAN	SE
Adam12	9.31	0.52	10.39	0.22	10.21	0.08	9.51	0.18	9.84	0.46	9.80	0.15
Adamts18	7.42	0.10	9.27	0.26	8.22	0.12	4.09	0.03	4.17	0.02	4.12	0.06
Alpl	10.57	0.39	11.63	0.26	11.70	0.12	11.06	0.26	11.37	0.49	11.37	0.21
Bmp3	8.62	0.29	9.39	0.30	9.66	0.13	8.57	0.04	8.86	0.39	8.83	0.15
Col12a1	11.71	0.59	12.09	0.20	12.70	0.10	12.14	0.15	11.62	0.37	12.14	0.20
Col6a3	9.55	0.53	9.87	0.28	10.59	0.09	10.16	0.18	9.65	0.36	10.34	0.20
Cpz	9.09	0.36	10.12	0.41	10.11	0.17	8.99	0.34	10.00	0.53	9.60	0.24
Dchs1	8.67	0.38	9.51	0.34	9.91	0.17	8.83	0.27	9.14	0.48	9.55	0.19
Ednra	7.47	0.13	8.35	0.21	8.59	0.19	7.35	0.13	8.26	0.22	8.17	0.23
Fat4	8.67	0.40	9.41	0.30	10.10	0.12	9.41	0.18	9.29	0.43	9.64	0.21
Fkbp10	10.42	0.42	11.39	0.28	11.49	0.12	10.47	0.28	10.84	0.49	10.86	0.21
Fkbp14	7.76	0.30	8.95	0.38	9.01	0.14	7.97	0.25	8.29	0.42	8.38	0.16
Fmod	8.04	0.08	8.64	0.26	10.72	0.24	9.62	0.20	8.51	0.16	9.93	0.33
Fndc3b	9.69	0.30	10.29	0.21	10.71	0.14	9.88	0.16	9.97	0.37	10.30	0.10
Fzd2	8.69	0.01	9.43	0.23	9.66	0.13	8.75	0.21	9.04	0.24	9.06	0.14
Grb10	8.45	0.30	9.40	0.29	9.65	0.18	8.71	0.22	9.00	0.42	9.12	0.17
Gtpbp4	6.75	0.63	5.91	0.17	5.49	0.07	5.44	0.15	5.05	0.15	4.98	0.11
Igsf10	8.59	0.31	9.39	0.28	9.95	0.21	9.01	0.34	9.40	0.51	9.67	0.26
Itga11	8.72	0.30	9.46	0.27	9.86	0.16	8.85	0.31	8.85	0.43	9.04	0.22
Lox	10.42	0.43	11.37	0.35	11.52	0.12	10.61	0.27	10.78	0.47	11.12	0.27
Mab21l2	7.05	0.44	7.85	0.24	8.09	0.13	7.07	0.29	7.75	0.45	7.50	0.16
Mdk	9.42	0.28	10.78	0.33	10.70	0.24	8.87	0.30	9.90	0.43	9.77	0.21
Mmp2	11.71	0.55	12.36	0.22	12.76	0.12	12.00	0.24	12.13	0.42	12.40	0.19
Mmp9	10.08	0.63	10.30	0.51	11.59	0.09	11.21	0.45	10.69	0.58	11.31	0.22
Myh10	6.99	0.11	7.78	0.25	8.08	0.20	7.17	0.16	7.71	0.35	7.69	0.12
Myl9	7.98	0.31	8.40	0.22	9.73	0.23	8.39	0.19	8.66	0.20	8.58	0.15
Mylk	7.58	0.43	8.59	0.18	8.20	0.11	7.38	0.12	8.01	0.31	7.66	0.12
Ncam1	8.63	0.35	9.47	0.20	10.16	0.21	8.80	0.29	9.45	0.45	9.35	0.17
Ostn	7.10	0.26	8.39	0.36	8.17	0.36	7.06	0.04	7.32	0.19	7.06	0.18
P4ha3	8.37	0.07	9.79	0.35	9.74	0.21	8.95	0.40	9.54	0.61	8.83	0.34
Panx3	6.95	0.03	9.53	0.36	8.15	0.15	6.85	0.36	7.79	0.50	7.38	0.13
Pcolce	10.43	0.53	11.21	0.32	11.53	0.13	10.70	0.25	11.01	0.39	11.09	0.18
Plod2	10.60	0.52	11.48	0.29	11.64	0.14	10.79	0.25	10.96	0.41	11.32	0.20
Postn	12.65	0.47	12.66	0.19	13.22	0.06	12.89	0.12	12.47	0.35	12.93	0.15
Prickle1	8.44	0.23	9.51	0.23	9.18	0.15	8.39	0.19	8.99	0.40	8.54	0.13
Prrx1	9.76	0.32	10.49	0.23	10.90	0.13	10.40	0.23	10.23	0.32	10.52	0.21
Pth1r	8.72	0.44	10.16	0.31	9.98	0.15	9.16	0.18	9.29	0.41	9.05	0.17
S100g	6.91	0.05	7.76	0.58	8.79	0.33	7.07	0.53	6.91	0.50	7.81	0.42
Sfrp4	9.51	0.32	10.61	0.37	10.82	0.15	10.09	0.31	10.25	0.71	10.35	0.23
Slc16a7	6.87	0.28	7.28	0.40	7.87	0.11	7.19	0.36	7.05	0.36	7.39	0.16
Tagln	8.52	0.65	8.63	0.28	10.47	0.32	9.23	0.23	8.90	0.30	9.15	0.21
Thbs2	10.41	0.43	11.30	0.25	11.57	0.15	10.81	0.16	11.08	0.52	11.06	0.25
Tmem119	8.50	0.31	9.50	0.30	9.69	0.18	8.49	0.12	8.65	0.34	8.74	0.15
Tnfrsf11b	7.45	0.35	7.93	0.36	8.64	0.13	7.66	0.14	7.72	0.30	7.73	0.16
Tnmd	5.95	0.09	7.30	0.21	6.96	0.20	5.60	0.11	5.70	0.12	5.27	0.08
Tnn	10.80	0.65	10.55	0.31	11.85	0.13	11.23	0.19	10.51	0.48	11.33	0.27
Vcan	7.90	0.25	9.15	0.26	9.24	0.21	7.95	0.20	8.74	0.20	8.90	0.28
