# Supplementary material for: Increased Cell Proliferation and Gene Expression of Genes Related to Bone Remodeling, Cell Adhesion and Collagen Metabolism in the Periodontal Ligament of Unopposed Molars in Growing Rats
Source: Front Physiol. 2017 Feb 10;8:75. doi: 10.3389/fphys.2017.00075 (PMC5301028; doi:10.3389/fphys.2017.00075)
Supplement: Supplementary file 2 [file Table2.PDF]

TABLE S2: Results of nanostring analysis. Linear values of the mean and standard error of the raw data.

	3 DAYS						15 DAYS					
	EO		EU		C		EO		EU		C	
	MEAN	SE	MEAN	SE	MEAN	SE	MEAN	SE	MEAN	SE	MEAN	SE
Adam12	3	2	1	0	1	0	2	0	1	0	4	2
Adamts18	502	91	1571	109	1098	185	870	174	1362	764	1166	198
Alpl	1018	214	1514	322	1815	266	1524	314	2101	822	1431	259
Bmp3	1784	399	2105	178	3404	552	2080	292	2401	623	2273	312
Col12a1	22075	2951	23846	805	52809	8958	37331	5884	22807	7675	33581	9864
Col6a3	13351	2805	13089	1069	28609	4024	30484	3626	21128	5775	29694	6183
Cpz	2007	763	2290	399	2491	343	1834	519	4139	1597	2168	286
Dchs1	608	179	806	197	1138	161	920	182	1093	415	1067	151
Ednra	1266	566	843	51	1595	291	834	136	1728	334	1443	273
Fat4	4246	789	4366	425	7550	977	7757	1539	6151	1799	7831	1015
Fkbp10	5203	1003	7222	634	10498	1517	7268	1717	9862	3390	8038	1214
Fkbp14	883	215	1319	206	1804	251	964	205	1467	564	1081	188
Fmod	632	365	1398	190	10329	2576	5719	683	1312	106	5414	1658
Fndc3b	5723	1297	6568	772	10290	1110	6684	946	7126	1737	7259	811
Fzd2	908	169	990	157	1208	132	1098	103	1247	259	1319	152
Grb10	2064	431	3271	608	4477	606	3087	642	3499	1082	3575	555
Gtpbp4	1340	163	1388	56	1420	93	1406	83	1268	163	1241	75
Igsf10	3578	418	4123	219	7944	1062	5324	1167	5714	1961	6492	1230
Itga11	2498	705	2491	374	3989	451	3690	453	3431	1089	4022	580
Lox	14691	2953	17963	728	37229	6044	24140	5516	26556	8283	26953	4667
Mab21l2	129	32	225	27	420	79	196	48	323	78	243	39
Mdk	4313	1188	7029	1132	8535	1327	4017	898	7692	2123	5512	1003
Mmp2	18581	3482	22559	3647	40204	5286	35296	6417	36598	11884	38094	6490
Mmp9	2063	532	2212	569	7300	437	6789	2007	5032	1870	5683	1303
Myh10	819	171	1146	163	2084	299	1058	219	1689	539	1382	219
Myl9	1198	377	920	127	3590	618	2465	722	1737	250	2285	319
Mylk	2874	1006	4810	660	5484	847	2869	574	4660	1324	2975	325
Ncam1	3158	990	4443	793	6422	908	3968	769	6653	1997	6335	731
Ostn	796	234	1568	72	840	126	644	114	581	172	431	105
P4ha3	201	65	315	82	243	45	228	61	247	104	146	42
Panx3	89	32	554	48	254	58	95	27	209	85	108	21
Pcolce	10431	1934	14722	1977	22861	3406	17346	3606	19945	5910	18144	2682
Plod2	12341	3443	15874	1288	31555	5638	16694	4182	20523	6348	21519	3454
Prickle1	1444	185	3262	280	3590	621	2012	362	3321	1091	1702	255
Prrx1	1729	378	2174	197	3329	413	2697	261	2031	417	2607	402
Pth1r	1338	430	2880	463	2793	385	1946	532	2471	784	1807	323
S100g	260	222	405	84	1667	962	380	186	275	134	748	217
Sfrp4	307	54	863	157	999	185	827	175	1201	508	804	195
Slc16a7	139	28	169	21	356	49	285	59	220	71	282	58
Tagln	3016	1107	2269	166	16508	3759	6971	1365	4812	1002	5816	935
Thbs2	3338	831	5996	1155	8389	1301	4292	878	7149	2964	4146	1121
Tmem119	2017	341	4469	425	9119	1983	3344	888	4207	1461	3876	737
Tnfrsf11b	411	124	442	96	1287	251	688	104	639	201	562	116
Tnmd	697	79	1302	222	1314	248	449	163	488	54	354	69
Tnn	16589	1575	11478	2149	54465	8571	47533	10775	28914	11162	48029	10881
Vcan	4332	2065	3430	236	5831	1186	2863	379	4594	953	4522	855
Zfp354c	785	241	866	122	1349	188	835	148	1086	371	972	136
